# Association of Cigarette Type Initially Smoked With Suicidal Behaviors Among Adolescents in Korea From 2015 to 2018

**DOI:** 10.1001/jamanetworkopen.2021.8803

**Published:** 2021-04-30

**Authors:** Seung Hoon Kim, Sung Hoon Jeong, Eun-Cheol Park, Sung-In Jang

**Affiliations:** 1 Department of Preventive Medicine, Yonsei University College of Medicine, Seoul, Republic of Korea; 2 Institute of Health Services Research, Yonsei University, Seoul, Republic of Korea; 3 Department of Public Health, Graduate School, Yonsei University, Seoul, Republic of Korea

## Abstract

**Importance:**

Assessment of whether past electronic cigarette (e-cigarette) use or initiating smoking with e-cigarettes is associated with suicidal behaviors among adolescents is needed to inform future research and public health interventions.

**Objective:**

To evaluate the association between starting smoking with e-cigarettes or conventional cigarettes and suicidal behaviors among Korean adolescents.

**Design, Setting, and Participants:**

This cross-sectional study analyzed data on adolescents in grades 7 through 12 who participated in the nationwide Korea Youth Risk Behavior Web-Based Survey between 2015 and 2018.

**Exposures:**

Type of cigarette initially smoked: electronic or conventional.

**Main Outcomes and Measures:**

Multiple logistic regression analysis was performed to examine the association between initial cigarette type and suicidal behaviors, including suicidal ideation and suicide planning and attempts. All participants completed questionnaires about their history of suicidal behavior and were categorized into groups according to the type of cigarette used at initiation of smoking and any subsequent change (or lack of change) in the type of cigarette used.

**Results:**

A total of 255 887 Korean adolescents (51.2% male; mean (SD) age, 15.0 [1.8] years) were included in the primary analysis. Among 131 094 male adolescents, 3310 boys (2.5%) initially used e-cigarettes and 27 368 boys (20.9%) initially used conventional cigarettes. Among 124 793 female adolescents, 952 girls (0.8%) initially used e-cigarettes and 9296 girls (7.4%) initially used conventional cigarettes. Of those who initially used e-cigarettes, 178 of 3310 boys (5.4%) and 134 of 952 girls (14.1%) attempted suicide. Of those who initially used conventional cigarettes, 946 of 27 368 boys (3.5%) and 911 of 9296 girls (9.8%) attempted suicide. Adolescents who initially used e-cigarettes had a higher risk of suicidal behaviors, including suicide planning (boys: adjusted odds ratio [AOR], 1.63 [95% CI, 1.40-1.89]; *P* < .001; girls: AOR, 1.55 [95% CI, 1.23-1.95]; *P* < .001) and suicide attempts (boys: AOR, 1.55 [95% CI, 1.28-1.87]; *P* < .001; girls, AOR, 1.64 [95% CI, 1.29-2.10]; *P* < .001) compared with those who initially used conventional cigarettes. Changing from e-cigarettes to conventional cigarettes was associated with a higher risk of suicide attempts among both boys (AOR, 1.89; 95% CI, 1.39-2.57; *P* < .001) and girls (AOR, 2.36; 95% CI, 1.53-3.64; *P* < .001) compared with changing from conventional cigarettes to e-cigarettes.

**Conclusions and Relevance:**

In this study, the initial use of e-cigarettes vs conventional cigarettes was associated with suicidal behaviors among adolescents. In future research on the association of e-cigarette use with adolescent mental health and interventions for suicide prevention, the type of cigarette initially used and changing the cigarette type should be considered.

## Key Points

**Question:**

Is the type of cigarette initially smoked associated with suicidal behaviors among adolescents in Korea?

**Findings:**

In this cross-sectional study of 255 887 Korean adolescents who participated in the nationwide Korea Youth Risk Behavior Web-Based Survey from 2015 to 2018, those who used electronic cigarettes when they started smoking had a higher risk of suicidal behavior than did those who used conventional cigarettes when they started smoking. Adolescents who changed from using electronic cigarettes to conventional cigarettes were more likely to exhibit suicidal behaviors than were those who changed from using conventional cigarettes to electronic cigarettes.

**Meaning:**

In this study, initial cigarette type was associated with suicidal behaviors among adolescents, which may have implications for public health policies and educational programs regarding electronic cigarette use.

## Teaser

This cross-sectional study uses data from the Korea Youth Risk Behavior Web-Based Survey to examine the association between the type of cigarette initially smoked and suicidal behaviors among Korean adolescents from 2015 to 2018.

## Introduction

Electronic cigarettes (e-cigarettes) are portable devices that deliver nicotine through the battery-powered vaporization of a solution of nicotine, propylene glycol, vegetable glycerin, and flavor chemicals. They are sometimes introduced as harmless or safer alternatives to conventional cigarettes. Although e-cigarettes deliver nicotine without the thousands of toxic substances contained in conventional cigarettes,^1,2^ the nicotine content of e-cigarettes can lead to addiction. It is unclear whether e-cigarettes are safer than conventional cigarettes because the toxic effects of e-cigarette components are not fully understood.

The use of e-cigarettes among adolescents has increased exponentially worldwide. In the US, current e-cigarette use among adolescents in grades 6 to 8 increased from 0.6% in 2011 to 4.9% in 2018; among adolescents in grades 9 to 12, e-cigarette use increased from 1.5% in 2011 to 20.8% in 2018.^3^ Among Korean adolescents, current conventional cigarette use increased from 6.4% (male: 9.5%; female: 3.1%) in 2017 to 6.7% (male: 9.3%; female: 3.8%) in 2019, and current e-cigarette use increased from 2.2% (male: 3.3%; female: 0.9%) in 2017 to 3.2% (male: 4.7%; female: 1.5%) in 2019.^4^ Statistics indicate a high smoking rate among boys and an increasing smoking rate among girls; thus, smoking among adolescents is a public health concern in South Korea.

South Korea has the highest suicide rate among the member countries of the Organization for Economic Cooperation and Development,^5^ and suicide is the leading cause of death among Korean adolescents. According to the Korea National Statistical Office, the suicide rate among youths increased from 7.7 per 100 000 people in 2017 to 9.1 per 100 000 people in 2018.^6,7^ Recognizing the factors associated with suicide is important; however, the differences in sociodemographic factors associated with suicidal behaviors, such as increased nonfatal suicidal behaviors among women and higher suicide rates among men,^8^ need to be considered. Of note, as part of the guidelines for clinical suicide assessment, physicians should specifically inquire about suicidal ideation and suicide planning and attempts.^9,10^

The association between smoking conventional cigarettes and mental health is well known.^11,12,13,14,15,16,17^ Previous studies,^11,12,13,14,15^ including longitudinal studies, have indicated that smoking during adolescence is associated with depression and suicidal behaviors, including suicidal ideation and suicide planning and attempts. However, most studies evaluating this association have focused on conventional cigarettes. Despite the increasing use of e-cigarettes and e-cigarette use involving inhaled nicotine, few studies have examined the association between using e-cigarettes (ie, vaping) and suicidal behaviors.^18,19^

Some studies^16,20^ have evaluated the association between e-cigarette use and mental health. A recent study^17^ of Korean adolescents found that youths who used both conventional cigarettes and e-cigarettes and those who used e-cigarettes only were more likely to exhibit suicidal ideation and suicide planning and attempts. Substances contained in e-cigarettes, such as e-liquids, are sometimes used in suicide attempts.^21,22^ However, most of these studies^16,19^ only examined the association between current or lifetime use of e-cigarettes and suicidal behaviors.

Adolescents have the highest rates of e-cigarette use, and unlike adults, they do not typically begin using e-cigarettes to quit smoking but are instead motivated by curiosity or appealing e-cigarette flavors.^20,23^ Furthermore, the distinct characteristics of adolescents who initially use e-cigarettes and their increased risk of subsequent use of conventional cigarettes^24,25,26^ warrants investigation into whether the initial use of e-cigarettes and the continuation of smoking using conventional cigarettes are associated with mental health. To our knowledge, no previous studies have investigated the association between the initial use of e-cigarettes and suicidal behaviors.

Therefore, we assessed the association between the type of cigarette initially used and suicidal behaviors among Korean adolescents. Furthermore, we examined whether switching the cigarette type after smoking initiation and the timing of the change were associated with suicidal behaviors.

## Methods

### Study Population and Data

Data for this cross-sectional study were obtained from the Korea Youth Risk Behavior Web-Based Survey (KYRBWS), which was conducted annually from 2015 to 2018 by the Korea Centers for Disease Control and Prevention (KCDC). Ethics approval for the KYRBWS was waived by the KCDC’s institutional review board in accordance with the Bioethics and Safety Act of 2015.^27^ All data used in this study are publicly available on the KYRBWS website.^4^ The KYRBWS complied with the Declaration of Helsinki,^28^ and all individuals who participated in the KYRBWS provided informed consent. This study followed the Strengthening the Reporting of Observational Studies in Epidemiology (STROBE) reporting guideline for cross-sectional studies.^29^

The KYRBWS was an anonymous, self-administered, structured questionnaire that had a complex research design, which included multistage sampling, stratification, and clustering. The KYRBWS used an online survey system that did not allow respondents to proceed to the next section of the questionnaire unless all questions were answered in the current section. Responses that had logical errors and responses that were outliers (eg, the respondent incorrectly stated that he or she was younger than 12 years, thus could not have been included in the KYRBWS because of its grade-level criteria) were processed as missing values. The questionnaire contained approximately 120 items in 15 categories, including demographic characteristics and health-associated behaviors. Students in grades 7 to 12 were the target population. The question, “When did you start vaping e-cigarettes?” was included in the KYRBWS from 2015 to 2018. Therefore, data from only these years (comprising 255 887 adolescent participants) were analyzed; there were no missing values.

### Variables

The main dependent variables were suicidal behaviors, comprising suicidal ideation, suicide planning, and suicide attempts. Suicidal ideation was assessed on the KYRBWS through the question, “Have you seriously considered suicide in the past 12 months?” Suicide planning was assessed by the question, “Did you make any specific plans for suicide in the past 12 months?” Suicide attempts were assessed by the question, “Have you attempted suicide in the past 12 months?” Suicidal ideation, suicide planning, and suicide attempts were categorized based on whether respondents answered these questions with yes or No. Each of the 3 questions about suicidal behaviors was composed of 3 separate variables (suicidal ideation, suicide planning, and suicide attempts).

The independent variable was the initial type of cigarette used, which was determined by yes or no answers to the questions, “Have you ever smoked even 1 puff in your lifetime? and “Have you ever used e-cigarettes?” Participants who answered no to both questions were classified into the group that had never smoked, and participants who answered yes to only 1 of the questions were classified into the group that started smoking using that type of cigarette (with the assumption that answering yes to question 1 and no to question 2 indicated use of conventional cigarettes). Those who answered yes to both questions were categorized according to the initial type of cigarette used and the school year (ie, grade) during which they started smoking either e-cigarettes or conventional cigarettes. The school year of smoking initiation was used to classify participants who initially used e-cigarettes into the group that started smoking with e-cigarettes, and participants who initially used conventional cigarettes or both conventional cigarettes and e-cigarettes during the same school year were classified into the group that started smoking with conventional cigarettes. This categorization allowed us to differentiate the association between initial use of e-cigarettes vs initial use of conventional cigarettes and suicidal behaviors.

Based on these categories, the independent variable was divided into 3 groups: never smoked, started with e-cigarettes, and started with conventional cigarettes. For the secondary analyses, we divided the group of participants who had smoked at least 1 e-cigarette or 1 conventional cigarette into subgroups based on whether they had changed the cigarette type used. The KYRBWS did not assess whether participants smoked both cigarette types after changing their initial cigarette type; thus, all participants who reported smoking a cigarette type different than the type they initially smoked were included in the group that switched cigarette type. For the subgroup analyses, participants who changed cigarette type were divided according to their current smoking status and whether they changed cigarette type within 2 years of starting to smoke that cigarette type.

The covariates were school grade (7-12), self-reported economic status (low, medium-low, medium, medium-high, or high), living situation (living with immediate family, with extended family, or without immediate or extended family), self-reported academic achievement (low, medium-low, medium, medium-high, or high), alcohol use (ever or never), physical activity level (low or high, with high indicating >20 minutes of intense physical activity ≥3 days per week), self-reported health status (low, medium, or high), and perceived stress level (low, medium, or high).^19,30^

### Statistical Analysis

All analyses were conducted separately by sex to account for sex-specific differences in smoking rates and suicidal behaviors.^4,8^ Differences in the frequency and proportion of categorical variables were evaluated using χ^2^ tests. Multiple logistic regression analysis was performed to examine the association between initial cigarette type and suicidal behaviors, with adjustment for covariates in the primary analyses. Adjusted odds ratios (AORs) and 95% CIs were calculated. Secondary analyses were performed to investigate the association between changing cigarette type and suicidal behaviors, and subgroup analyses were conducted to evaluate the association between the timing of the change in cigarette type and suicidal behaviors. All statistical analyses were performed using SAS software, version 9.4 (SAS Institute), and a weighted logistic regression procedure was used to account for the complex and stratified sampling design. Bonferroni correction was applied to handle type 1 errors owing to multiple comparisons, with a 2-sided significance threshold of *P* < .02 (number of contrasts, 0.05/3 = 0.016) for the primary analysis and *P* < .002 for the secondary and subgroup analyses (number of contrasts, 0.5/21 = 0.0024).

## Results

The primary analysis included 255 887 adolescents (131 094 [51.2%] male; mean [SD] age, 15.0 [1.8] years). Among male participants, 3310 (2.5%) initially used e-cigarettes, 27 368 (20.9%) initially used conventional cigarettes, and 100 416 (76.6%) never smoked. Among female participants, 952 (0.8%) initially used e-cigarettes, 9296 (7.4%) initially used conventional cigarettes, and 114 545 (91.8%) never smoked. Among male participants, rates of suicidal ideation (500 [15.1%]), suicide planning (271 [8.2%]), and suicide attempts (178 [5.4%]) were higher among those who initially used e-cigarettes compared with those who initially used conventional cigarettes (3709 [13.6%], 1477 [5.4%], and 946 [3.5%], respectively) and those who never smoked (8062 [8.0%], 2867 [2.9%], and 1489 [1.5%], respectively). Among female adolescents who initially used e-cigarettes, rates of suicidal behaviors were also higher (suicidal ideation: 279 [29.3%]; suicide planning: 146 [15.3%]; suicide attempts: 134 [14.1%]) than rates among those who initially used conventional cigarettes (2635 [28.3%], 1040 [11.2%], and 911 [9.8%], respectively) and those who never smoked (16 082 [14.0%], 4354 [4.0%], and 3041 [2.7], respectively) (Table 1).

**Table 1.** Characteristics of the 255 887 Korean Adolescents Included in the Analysis

Characteristic	Suicidal ideation						Suicide planning						Suicide attempt					
	Male participants			Female participants			Male participants			Female participants			Male participants			Female participants		
	Total, No.	Exhibited suicidal ideation, No. (%)	*P* value	Total, No.	Exhibited suicidal ideation, No. (%)	*P* value	Total, No.	Exhibited suicide planning, No. (%)	*P* value	Total, No.	Exhibited suicide planning, No. (%)	*P* value	Total, No.	Exhibited suicide attempt, No. (%)	*P* value	Total, No.	Exhibited suicide attempt, No. (%)	*P* value
Total participants, No.	131 094	12 271 (9.4)	NA	124 793	18 807 (15.1)	NA	131 094	4615 (3.5)	NA	124 793	5720 (4.6)	NA	131 094	2613 (2.0)	NA	124 793	4086 (3.3)	NA
Type of cigarette used at initiation of smoking																		
Electronic	3310	500 (15.1)	<.001	952	279 (29.3)	<.001	3310	271 (8.2)	<.001	952	146 (15.3)	<.001	3310	178 (5.4)	<.001	952	134 (14.1)	<.001
Conventional	27 368	3709 (13.6)		9296	2635 (28.3)		27 368	1477 (5.4)		9296	1040 (11.2)		27 368	946 (3.5)		9296	911 (9.8)	
Never smoked	100 416	8062 (8.0)		114 545	16 082 (14.0)		100 416	2867 (2.9)		114 545	4534 (4.0)		100 416	1489 (1.5)		114 545	3041 (2.7)	
School grade																		
7	21 230	1706 (8.0)	<.001	20 075	2907 (14.5)	<.001	21 230	698 (3.3)	<.001	20 075	1001 (5.0)	<.001	21 230	397 (1.9)	<.001	20 075	806 (4.0)	<.001
8	21 913	2095 (9.6)		20 515	3487 (17.0)		21 913	802 (3.7)		20 515	1152 (5.6)		21 913	467 (2.1)		20 515	847 (4.1)	
9	22 437	2181 (9.7)		21 462	3470 (16.2)		22 437	886 (3.9)		21 462	1111 (5.2)		22 437	489 (2.2)		21 462	774 (3.6)	
10	21 520	1910 (8.9)		20 382	2862 (14.0)		21 520	665 (3.1)		20 382	765 (3.8)		21 520	367 (1.7)		20 382	574 (2.8)	
11	22 241	2208 (9.9)		20 781	3199 (15.4)		22 241	766 (3.4)		20 781	835 (4.0)		22 241	421 (1.9)		20 781	563 (2.7)	
12	21 753	2171 (10.0)		21 578	3071 (14.2)		21 753	798 (3.7)		21 578	856 (4.0)		21 753	472 (2.2)		21 578	522 (2.4)	
Economic status																		
Low	3928	872 (22.2)	<.001	3233	1018 (31.5)	<.001	3928	413 (10.5)	<.001	3233	433 (13.4)	<.001	3928	279 (7.1)	<.001	3233	335 (10.4)	<.001
Medium-low	15 491	2037 (13.1)		16 044	3601 (22.4)		15 491	687 (4.4)		16 044	1129 (7.0)		15 491	380 (2.5)		16 044	814 (5.1)	
Medium	57 982	4711 (8.1)		61 426	8384 (13.6)		57 982	1581 (2.7)		61 426	2301 (3.7)		57 982	850 (1.5)		61 426	1682 (2.7)	
Medium-high	37 800	3102 (8.2)		34 283	4652 (13.6)		37 800	1143 (3.0)		34 283	1347 (3.9)		37 800	607 (1.6)		34 283	913 (2.7)	
High	15 893	1549 (9.7)		9807	1341 (13.7)		15 893	791 (5.0)		9807	510 (5.2)		15 893	497 (3.1)		9807	342 (3.5)	
Living situation																		
With extended family	1131	252 (22.3)	<.001	848	255 (30.1)	<.001	1131	156 (13.8)	<.001	848	114 (13.4)	<.001	1131	92 (8.1)	<.001	848	87 (10.3)	<.001
With immediate family	124 001	11 243 (9.1)		118 871	17 894 (15.1)		124 001	4067 (3.3)		118 871	5289 (4.4)		124 001	2253 (1.8)		118 871	3762 (3.2)	
Without extended or immediate family	5962	776 (13.0)		5074	847 (16.7)		5962	392 (6.6)		5074	317 (6.2)		5962	268 (4.5)		5074	237 (4.7)	
Academic achievement																		
Low	14 292	1952 (13.7)	<.001	11 671	2713 (23.2)	<.001	14 292	764 (5.3)	<.001	11 671	970 (8.3)	<.001	14 292	521 (3.6)	<.001	11 671	820 (7.0)	<.001
Medium-low	28 883	2963 (10.3)		29 254	5095 (17.4)		28 883	1054 (3.6)		29 254	1499 (5.1)		28 883	556 (1.9)		29 254	1122 (3.8)	
Medium	36 011	3033 (8.4)		36 903	4932 (13.4)		36 011	1151 (3.2)		36 903	1397 (3.8)		36 011	593 (1.6)		36 903	961 (2.6)	
Medium-high	32 233	2596 (8.1)		32 739	4345 (13.3)		32 233	881 (2.7)		32 739	1232 (3.8)		32 233	442 (1.4)		32 739	793 (2.4)	
High	19 675	1727 (8.8)		14 226	1911 (13.4)		19 675	765 (3.9)		14 226	622 (4.4)		19 675	501 (2.5)		14 226	390 (2.7)	
Alcohol use																		
Ever	57 494	6843 (11.9)	<.001	43 625	8998 (20.6)	<.001	57 494	2606 (4.5)	<.001	43 625	2897 (6.6)	<.001	57 494	1549 (2.7)	<.001	43 625	2227 (5.1)	<.001
Never	73 600	5428 (7.4)		81 168	9998 (12.3)		73 600	2009 (2.7)		81 168	2823 (3.5)		73 600	1064 (1.4)		81 168	1859 (2.3)	
Physical activity level																		
Low	53 224	5081 (9.5)	.06	84 362	12 224 (14.5)	<.001	53 224	1729 (3.2)	<.001	84 362	3443 (4.1)	<.001	53 224	963 (1.8)	<.001	84 362	2363 (2.8)	<.001
High	77 870	7190 (9.2)		40 431	6772 (16.7)		77 870	2886 (3.7)		40 431	2277 (5.6)		77 870	1650 (2.1)		40 431	1723 (4.3)	
Self-reported health status																		
High	101 009	7378 (7.3)	<.001	83 994	9322 (11.1)	<.001	101 009	2878 (2.8)	<.001	83 994	2602 (3.1)	<.001	101 009	1522 (1.5)	<.001	83 994	1863 (2.2)	<.001
Medium	23 570	3239 (13.7)		31 493	6361 (20.2)		23 570	1089 (4.6)		31 493	1888 (6.0)		23 570	659 (2.8)		31 493	1395 (4.4)	
Low	6515	1654 (25.4)		9306	3313 (35.6)		6515	648 (9.9)		9306	1230 (13.2)		6515	432 (6.6)		9306	828 (8.9)	
Perceived stress level																		
Low	33 654	787 (2.3)	<.001	17 579	500 (2.8)	<.001	33 654	570 (1.7)	<.001	17 579	246 (1.4)	<.001	33 654	341 (1.0)	<.001	17 579	160 (0.9)	<.001
Medium	57 708	2848 (4.9)		51 071	3337 (6.5)		57 708	1148 (2.0)		51 071	903 (1.8)		57 708	570 (1.0)		51 071	645 (1.3)	
High	39 732	8636 (21.7)		56 143	15 159 (27.0)		39 732	2897 (7.3)		56 143	4571 (8.1)		39 732	1702 (4.3)		56 143	3281 (5.8)	

Abbreviation: NA, not applicable.

Participants who started smoking using either type of cigarette were more likely to attempt suicide than were those who never smoked. Among boys who initiated smoking with e-cigarettes, the AOR was 2.90 (95% CI, 2.40-3.51; *P* < .001), and among those who initiated smoking with conventional cigarettes, the AOR was 1.88 (95% CI, 1.68-2.09; *P* < .001). Among girls who initiated smoking with e-cigarettes, the AOR was 4.04 (95% CI, 3.20-5.09; *P* < .001), and among those who initiated smoking with conventional cigarettes, the AOR was 2.46 (95% CI, 2.23-2.71; *P* < .001). Other factors associated with suicidal behaviors are shown in Table 2.

**Table 2.** Factors Associated With Suicidal Behaviors

Variable	Suicidal ideation				Suicide planning				Suicide attempt			
	Male participants		Female participants		Male participants		Female participants		Male participants		Female participants	
	AOR (95% CI)	*P* value	AOR (95% CI)	*P* value	AOR (95% CI)	*P* value	AOR (95% CI)	*P* value	AOR (95% CI)	*P* value	AOR (95% CI)	*P* value
Type of cigarette used at initiation of smoking												
Electronic	1.74 (1.55-1.96)	<.001	1.83 (1.53-2.21)	<.001	2.54 (2.19-2.95)	<.001	2.94 (2.35-3.67)	<.001	2.90 (2.40-3.51)	<.001	4.04 (3.20-5.09)	<.001
Conventional	1.38 (1.31-1.46)	<.001	1.54 (1.44-1.64)	<.001	1.56 (1.43-1.70)	<.001	1.90 (1.74-2.08)	<.001	1.88 (1.68-2.09)	<.001	2.46 (2.23-2.71)	<.001
Never smoked	1 [Reference]	NA	1 [Reference]	NA	1 [Reference]	NA	1 [Reference]	NA	1 [Reference]	NA	1 [Reference]	NA
School grade												
7	2.04 (1.86-2.23)	<.001	1.96 (1.82-2.11)	<.001	1.50 (1.32-1.71)	<.001	2.62 (2.31-2.96)	<.001	1.65 (1.39-1.96)	<.001	3.98 (3.44-4.59)	<.001
8	1.98 (1.82-2.17)	<.001	2.05 (1.91-2.21)	<.001	1.50 (1.32-1.70)	<.001	2.49 (2.21-2.80)	<.001	1.64 (1.40-1.92)	<.001	3.27 (2.84-3.76)	<.001
9	1.83 (1.68-2.00)	<.001	1.80 (1.67-1.93)	<.001	1.57 (1.39-1.77)	<.001	2.08 (1.85-2.34)	<.001	1.54 (1.32-1.81)	<.001	2.49 (2.18-2.86)	<.001
10	1.14 (1.04-1.24)	.01	1.29 (1.21-1.38)	<.001	1.03 (0.91-1.16)	.67	1.25 (1.11-1.41)	<.001	0.97 (0.83-1.14)	.74	1.58 (1.37-1.82)	<.001
11	1.08 (1.00-1.17)	.02	1.21 (1.13-1.30)	<.001	1.02 (0.91-1.15)	.70	1.13 (1.01-1.26)	.03	0.97 (0.83-1.13)	.67	1.20 (1.04-1.38)	.007
12	1 [Reference]	NA	1 [Reference]	NA	1 [Reference]	NA	1 [Reference]	NA	1 [Reference]	NA	1 [Reference]	NA
Economic status												
Low	1.43 (1.28-1.60)	<.001	1.59 (1.41-1.79)	<.001	1.34 (1.15-1.56)	<.001	1.51 (1.26-1.81)	<.001	1.22 (1.02-1.47)	.03	1.54 (1.27-1.87)	<.001
Medium-low	0.94 (0.87-1.03)	.19	1.21 (1.11-1.32)	<.001	0.69 (0.61-0.78)	<.001	0.93 (0.81-1.07)	.32	0.64 (0.55-0.76)	<.001	0.97 (0.84-1.13)	.73
Medium	0.74 (0.68-0.79)	<.001	0.85 (0.79-0.91)	<.001	0.54 (0.49-0.60)	<.001	0.66 (0.58-0.74)	<.001	0.51 (0.44-0.57)	<.001	0.71 (0.62-0.81)	<.001
Medium-high	0.80 (0.74-0.86)	<.001	0.93 (0.86-1.00)	.06	0.62 (0.56-0.69)	<.001	0.73 (0.64-0.82)	<.001	0.58 (0.51-0.66)	<.001	0.77 (0.67-0.89)	<.001
High	1 [Reference]	NA	1 [Reference]	NA	1 [Reference]	NA	1 [Reference]	NA	1 [Reference]	NA	1 [Reference]	NA
Living situation												
With extended family	1.73 (1.40-2.14)	<.001	1.57 (1.27-1.93)	<.001	1.89 (1.48-2.42)	<.001	1.44 (1.08-1.92)	.01	1.49 (1.10-2.03)	.01	1.31 (0.95-1.81)	.11
With immediate family	0.69 (0.62-0.76)	<.001	0.88 (0.79-0.97)	.009	0.48 (0.42-0.55)	<.001	0.60 (0.52-0.70)	<.001	0.41 (0.35-0.48)	<.001	0.60 (0.50-0.70)	<.001
Without extended or immediate family	1 [Reference]	NA	1 [Reference]	NA	1 [Reference]	NA	1 [Reference]	NA	1 [Reference]	NA	1 [Reference]	NA
Academic achievement												
Low	1.09 (0.99-1.19)	.07	1.21 (1.12-1.31)	<.001	1.05 (0.93-1.19)	.40	1.23 (1.08-1.40)	.002	1.08 (0.93-1.26)	.30	1.68 (1.45-1.93)	<.001
Medium-low	1.06 (0.98-1.14)	.15	1.13 (1.05-1.21)	.001	0.95 (0.85-1.06)	.39	1.03 (0.92-1.15)	.65	0.75 (0.66-0.87)	<.001	1.24 (1.09-1.41)	.001
Medium	1.01 (0.93-1.05)	.85	0.97 (0.90-1.04)	.36	0.99 (0.88-1.10)	.79	0.91 (0.81-1.01)	.08	0.80 (0.69-0.92)	.001	1.03 (0.90-1.18)	.66
Medium-high	0.95 (0.88-1.02)	.14	1.00 (0.93-1.07)	.93	0.80 (0.71-0.89)	<.001	0.92 (0.83-1.03)	.15	0.64 (0.56-0.73)	<.001	1.01 (0.89-1.15)	.89
High	1 [Reference]	NA	1 [Reference]	NA	1 [Reference]	NA	1 [Reference]	NA	1 [Reference]	NA	1 [Reference]	NA
Alcohol use												
Ever	1.36 (1.30-1.43)	<.001	1.53 (1.47-1.60)	<.001	1.30 (1.20-1.40)	<.001	1.54 (1.43-1.65)	<.001	1.38 (1.24-1.52)	<.001	1.77 (1.63-1.93)	<.001
Never	1 [Reference]	NA	1 [Reference]	NA	1 [Reference]	NA	1 [Reference]	NA	1 [Reference]	NA	1 [Reference]	NA
Physical activity level												
Low	0.91 (0.86-0.95)	<.001	0.82 (0.79-0.85)	<.001	0.79 (0.73-0.85)	<.001	0.75 (0.70-0.80)	<.001	0.75 (0.68-0.83)	<.001	0.71 (0.66-0.76)	<.001
High	1 [Reference]	NA	1 [Reference]	NA	1 [Reference]	NA	1 [Reference]	NA	1 [Reference]	NA	1 [Reference]	NA
Self-reported health status												
High	0.39 (0.37-0.43)	<.001	0.38 (0.36-0.40)	<.001	0.39 (0.35-0.43)	<.001	0.33 (0.30-0.36)	<.001	0.32 (0.28-0.37)	<.001	0.36 (0.33-0.40)	<.001
Medium	0.59 (0.54-0.64)	<.001	0.58 (0.55-0.62)	<.001	0.53 (0.47-0.60)	<.001	0.51 (0.47-0.56)	<.001	0.51 (0.44-0.59)	<.001	0.59 (0.53-0.65)	<.001
Low	1 [Reference]	NA	1 [Reference]	NA	1 [Reference]	NA	1 [Reference]	NA	1 [Reference]	NA	1 [Reference]	NA
Perceived stress level												
Low	0.11 (0.10-0.12)	<.001	0.10 (0.09-0.11)	<.001	0.27 (0.25-0.30)	<.001	0.21 (0.18-0.25)	<.001	0.30 (0.26-0.34)	<.001	0.20 (0.17-0.24)	<.001
Medium	0.22 (0.21-0.23)	<.001	0.23 (0.22-0.24)	<.001	0.31 (0.29-0.34)	<.001	0.27 (0.25-0.29)	<.001	0.29 (0.26-0.32)	<.001	0.28 (0.25-0.31)	<.001
High	1 [Reference]	NA	1 [Reference]	NA	1 [Reference]	NA	1 [Reference]	NA	1 [Reference]	NA	1 [Reference]	NA

Abbreviations: AOR, adjusted odds ratio; NA, not applicable.

**Figure 1.**
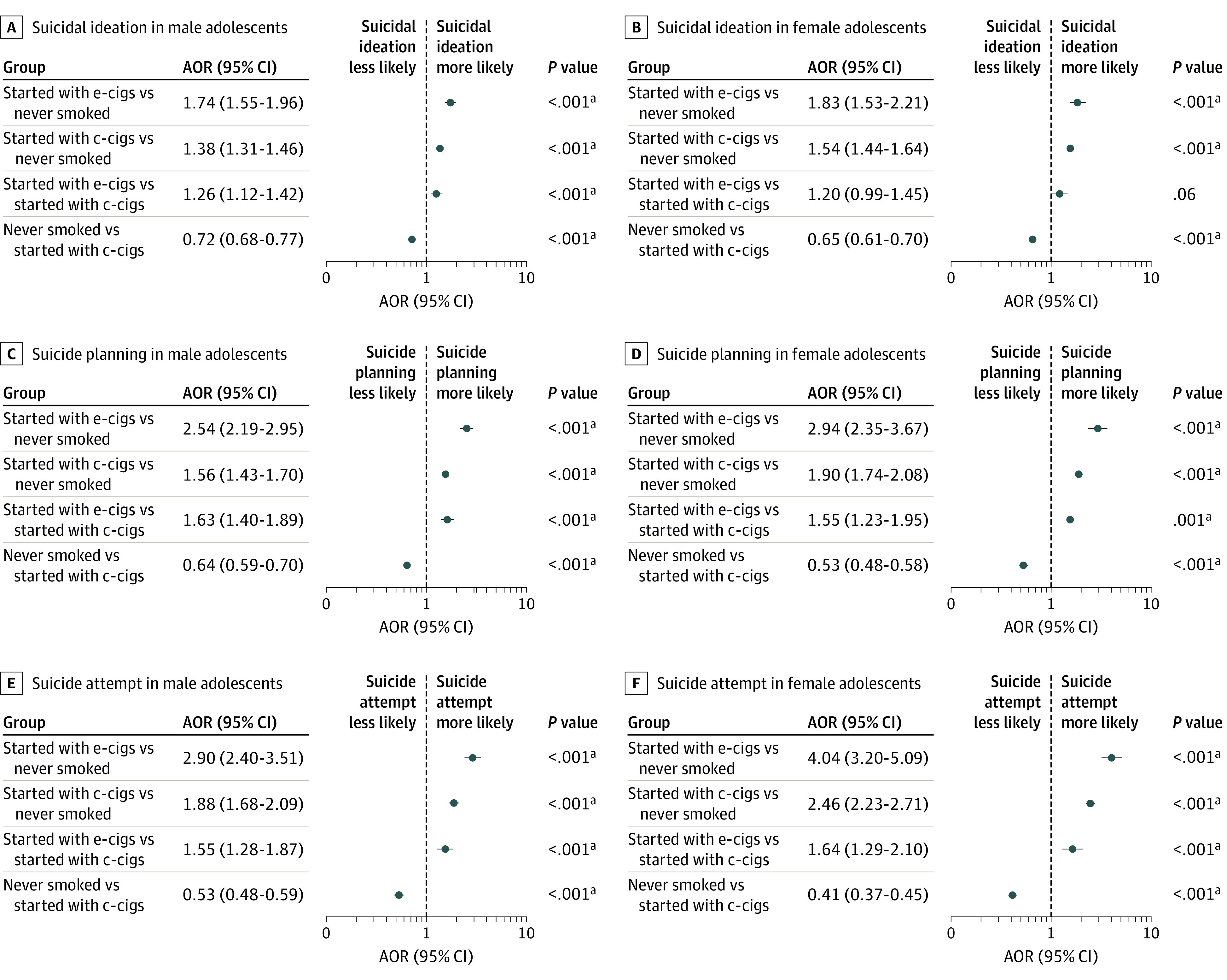
Association Between Initial Type of Cigarette Smoked and Suicidal Behaviors Analyses were adjusted for school grade, economic status, living situation, academic achievement, alcohol use, physical activity level, self-reported health status, and perceived stress level. AOR indicates adjusted odds ratio; c-cigs, conventional cigarettes; and e-cigs, electronic cigarettes. ^a^Statistically significant after applying Bonferroni correction.

After adjusting for covariates, male adolescents who initially used e-cigarettes had a higher risk of suicidal ideation (AOR, 1.26; 95% CI, 1.12-1.42; *P* < .001), suicide planning (AOR, 1.63; 95% CI, 1.40-1.89; *P* < .001), and suicide attempts (AOR, 1.55; 95% CI, 1.28-1.87; *P* < .001) compared with those who initially used conventional cigarettes. Among female adolescents, the initial use of e-cigarettes was significantly associated with suicide planning (AOR, 1.55; 95% CI, 1.23-1.95; *P* < .001) and suicide attempts (AOR, 1.64; 95% CI, 1.29-2.10; *P* < .001) compared with the initial use of conventional cigarettes (Figure 1).

The demographic characteristics of participants included in the secondary analyses are presented in eTable 1 in the Supplement. Results of the χ^2^ test for changing cigarette type and covariates were statistically significant. The risk of suicidal ideation, suicide planning, and suicide attempts increased when the cigarette type changed, especially when the change was from e-cigarettes to conventional cigarettes (Table 3). The highest AOR for suicide attempts was 6.91 (95% CI, 4.62-10.33; *P* < .001) among female adolescents who initially used e-cigarettes and changed to conventional cigarettes. Adolescents of both sexes who initially used e-cigarettes and changed to conventional cigarettes had a higher risk of suicidal behavior than did those who initially used conventional cigarettes and changed to e-cigarettes. Among boys, the AOR for suicidal ideation was 1.88 (95% CI, 1.51-2.34; *P* < .001); for suicide planning, 2.29 (95% CI, 1.76-2.97; *P* < .001); and for suicide attempts, 1.89 (95% CI, 1.39-2.57; *P* < .001). Among girls, the AOR for suicidal ideation was 1.84 (95% CI, 1.24-2.71; *P* = .002); for suicide planning, 2.22 (95% CI, 1.47-3.36; *P* = .001); and for suicide attempts, 2.36 (95% CI, 1.53-3.64; *P* < .001). Statistical significance remained after Bonferroni correction.

**Table 3.** Association Between Change in Cigarette Type and Suicidal Behaviors^a^

Variable	Suicidal ideation				Suicide planning				Suicide attempt			
	Male participants		Female participants		Male participants		Female participants		Male participants		Female participants	
	AOR (95% CI)	*P* value	AOR (95% CI)	*P* value	AOR (95% CI)	*P* value	AOR (95% CI)	*P* value	AOR (95% CI)	*P* value	AOR (95% CI)	*P* value
Never smoking as reference												
Switched from e-cigarettes to conventional cigarettes (or used both after switching)	2.54 (2.06-3.13)	<.001^b^	2.75 (1.89-4.01)	<.001^b^	3.56 (2.77-4.58)	<.001^b^	4.58 (3.11-6.75)	<.001^b^	4.05 (3.03-5.43)	<.001^b^	6.91 (4.62-10.33)	<.001^b^
Initiated smoking using both e-cigarettes and conventional cigarettes (in same year)	1.64 (1.48-1.82)	<.001^b^	1.91 (1.61-2.25)	<.001^b^	1.99 (1.73-2.29)	<.001^b^	2.85 (2.30-3.53)	<.001^b^	2.48 (2.09-2.95)	<.001^b^	3.63 (1.53-2.16)	<.001^b^
Switched from conventional cigarettes to e-cigarettes (or used both after switching)	1.35 (1.24-1.48)	<.001^b^	1.50 (1.32-1.70)	<.001^b^	1.56 (1.38-1.77)	<.001^b^	2.06 (1.75-2.42)	<.001^b^	2.14 (1.84-2.50)	<.001^b^	2.93 (2.48-3.47)	<.001^b^
Used only e-cigarettes	1.46 (1.27-1.68)	<.001^b^	1.57 (1.26-1.95)	<.001^b^	2.15 (1.79-2.57)	<.001^b^	2.38 (1.82-3.12)	<.001^b^	2.47 (1.96-3.11)	<.001^b^	3.09 (2.31-4.15)	<.001^b^
Used only conventional cigarettes	1.33 (1.25-1.43)	<.001^b^	1.50 (1.39-1.62)	<.001^b^	1.45 (1.30-1.61)	<.001^b^	1.71 (1.53-1.91)	<.001^b^	1.55 (1.34-1.78)	<.001^b^	2.13 (1.89-2.40)	<.001^b^
Never smoked	1 [Reference]	NA	1 [Reference]	NA	1 [Reference]	NA	1 [Reference]	NA	1 [Reference]	NA	1 [Reference]	NA
Switching from conventional cigarettes to e-cigarettes as reference												
Switched from e-cigarettes to conventional cigarettes (or used both after switching)	1.88 (1.51-2.34)	<.001^b^	1.84 (1.24-2.71)	.002^b^	2.29 (1.76-2.97)	<.001^b^	2.22 (1.47-3.36)	<.001^b^	1.89 (1.39-2.57)	<.001^b^	2.36 (1.53-3.64)	<.001^b^
Initiated smoking using both e-cigarettes and conventional cigarettes (in same year)	1.21 (1.07-1.37)	.002^b^	1.27 (1.03-1.57)	.02	1.28 (1.08-1.51)	.004	1.38 (1.06-1.80)	.02	1.16 (0.95-1.42)	.16	1.24 (0.95-1.62)	.12
Switched from conventional cigarettes to e-cigarettes (or used both after switching)	1 [Reference]	NA	1 [Reference]	NA	1 [Reference]	NA	1 [Reference]	NA	1 [Reference]	NA	1 [Reference]	NA

Abbreviations: AOR, adjusted odds ratio; NA, not applicable.

^a^ Adjusted for school grade, economic status, living situation, academic achievement, alcohol use, physical activity level, self-reported health status, and perceived stress level.

^b^ Statistically significant after applying Bonferroni correction.

**Figure 2.**
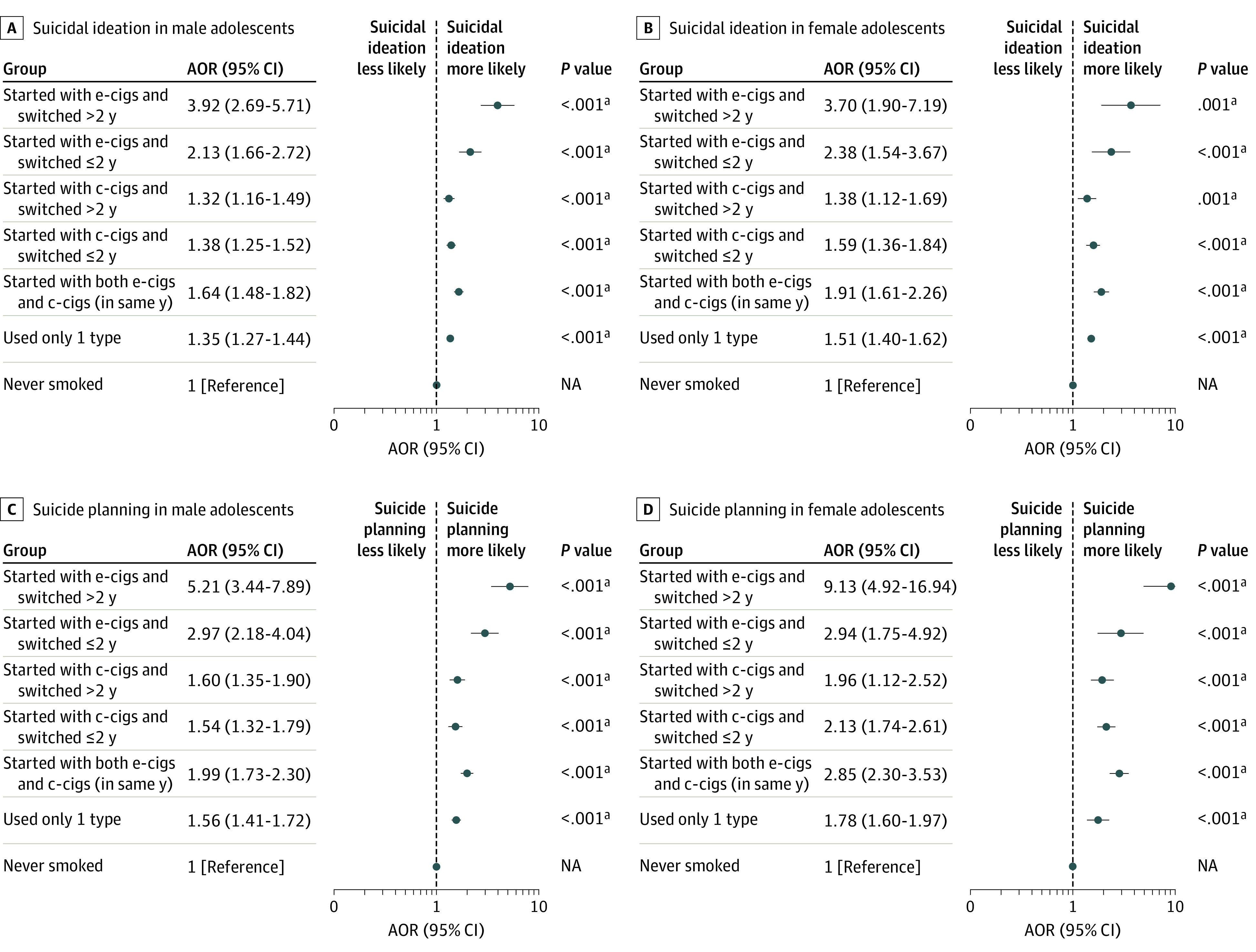
Association Between Timing of Change in Cigarette Type and Suicidal Behaviors Analyses were adjusted for school grade, economic status, living situation, academic achievement, alcohol use, physical activity level, self-reported health status, and perceived stress level. AOR indicates adjusted odds ratio; c-cigs, conventional cigarettes; e-cigs, electronic cigarettes; and NA, not applicable. ^a^Statistically significant after applying Bonferroni correction.

Figure 2 shows the subgroup analyses of the timing in change of cigarette type. Switching from e-cigarettes to conventional cigarettes after 2 years had the strongest association with suicidal ideation among boys (AOR, 3.92; 95% CI, 2.69-5.71; *P* < .001) and among girls (AOR, 3.70; 95% CI, 1.90-7.19; *P* = .001) and with suicide planning among boys (AOR, 5.21; 95% CI, 3.44-7.89; *P* < .001) and among girls (AOR, 9.13; 95% CI, 4.92-16.94; *P* < .001). The association between initial cigarette type and suicidal behaviors stratified by current smoking status is presented in eTable 2 in the Supplement.

Two sensitivity analyses were conducted to confirm the robustness of the results. Similar results were found with the exception of adolescents who started smoking or changed cigarette type within 12 months of completing the survey. In the analysis conducted using age rather than school grade as a covariate, similar results were observed (eTables 3-5 in the Supplement).

## Discussion

To our knowledge, this study is the first to identify an association between cigarette type initially smoked and suicidal behaviors using multiyear national survey data (2015-2018). Furthermore, the data were obtained from approximately 250 000 respondents of a national survey based on random cluster sampling,^31^ which ensured that the results were sufficiently representative of Korean adolescents. Our findings were similar to those of previous studies^17,25^ indicating that adolescents who had ever smoked were more likely to show suicidal behaviors than those who had never smoked.

Of note, adolescents who initially used e-cigarettes had a higher risk of suicide planning and suicide attempts than did those who initially used conventional cigarettes. In the secondary and subgroup analyses, the group that initially used e-cigarettes and switched to using conventional cigarettes was the most likely to exhibit suicidal behaviors. Furthermore, switching from e-cigarettes to conventional cigarettes after 2 years had the strongest association with suicidal ideation and suicide planning compared with changing from smoking e-cigarettes to conventional cigarettes within 2 years. Among male adolescents who currently smoked, those who initially used e-cigarettes had a higher likelihood of suicidal ideation, suicide planning, and suicide attempts.

Adolescents who use e-cigarettes before conventional cigarettes have been reported to have better school performance and higher intake of caffeinated drinks.^32^ However, to our knowledge, no studies have examined the association between the use of e-cigarettes before conventional cigarettes and mental health.

The present findings may be explained by several biological and behavioral mechanisms. E-cigarette aerosol contains highly oxidizing free-base nicotine, which is easily absorbed by the body and is the most addictive form of nicotine.^33,34,35^ Even nicotine-free e-cigarettes contain nicotine in their aerosols, and discrepancies between the labeled and actual amounts of nicotine have been found.^36,37^ Continuous exposure to nicotine may reduce serotonin and its metabolites, which can diminish the protection that serotonin provides from stress-induced processes that cause disease.^38,39,40^ In addition, prolonged nicotine administration can disrupt the brain's dopamine pathway, amplify sensitivity to stress, and distort mechanisms that alleviate depressive symptoms.^41^

Given that the nicotine concentration in e-cigarettes is generally lower than that in conventional cigarettes,^42^ nicotine withdrawal phenomenon or nicotine dependence can also occur in adolescents who initially use e-cigarettes. This withdrawal or dependence may lead adolescents to seek other nicotine-containing products, such as conventional cigarettes (the gateway theory^24,26^), and further increase their impulsivity.^24,26,43^ Furthermore, the different efficacy of nicotine vaporization and the perception that e-cigarettes are safer and healthier than conventional cigarettes may encourage adolescents to smoke e-cigarettes more frequently, resulting in excessive nicotine exposure.^42,44^ Substances in e-liquids may also alter the brains of adolescents and may be associated with suicidal behaviors. The flavor chemicals in e-liquids, which are not used in conventional cigarettes, are associated with increases in inflammatory cytokines, such as interleukin 1β and interleukin 6, which have important roles in the pathophysiology of suicidal behaviors.^45,46^ In addition, through the use of e-cigarettes, adolescents can absorb various trace metals, including arsenic, aluminum, and lead, which can affect the central and peripheral nervous systems and mental health.^47,48,49^ E-cigarette components, such as e-liquids, flavoring, and metallic coils, also provoke oxidative stress, which harms developing brains.^50^ Therefore, initial or current use of e-cigarettes may be associated with aggressive and impulsive behavior, impaired cognition, and increased suicidal ideation. Although it is difficult to acquire standardized toxicologic data regarding e-cigarettes,^51^ future studies are warranted to investigate the molecular composition of the toxicity profile of e-cigarettes.

The distinct characteristics of adolescents who initially use e-cigarettes may also be associated with suicidal behaviors. Adolescents who first smoked with e-cigarettes have lower levels of rebelliousness, and they may be sensitive to nicotine or nicotine withdrawal.^43,52,53^ Moreover, e-cigarette users have an increased risk of other drug use and mental health problems, including attention-deficit/hyperactivity disorder, anxiety, low self-esteem, and high impulsivity.^54,55^ The toxicity of e-cigarettes, in conjunction with the aforementioned characteristics of adolescents who used e-cigarettes before conventional cigarettes, may have been associated with increases in suicidal behavior.

Owing to the cross-sectional nature of this study, we could not establish causality or determine the direction of the association. However, we used all applicable measures to estimate the direction of the association, including adjustment for sociodemographic characteristics and established factors associated with adolescent suicidal behaviors, and conducted sensitivity analyses that excluded adolescents who started smoking or changed cigarette type within 12 months of the KYRBWS evaluation period for suicidal behaviors.

Our findings suggest that caution should be taken regarding the risk of suicidal behaviors associated with the initial use of e-cigarettes and the subsequent change in cigarette type among adolescents; this is an important hypothesis to be confirmed in future longitudinal studies. If longitudinal clinical trials are able to determine causality, the findings may indicate that e-cigarettes are as unhealthy and unsafe as conventional cigarettes, at least with regard to suicidal behaviors among adolescents. The findings may also justify changes in public health policy and practice, such as the inclusion of questions about e-cigarette history when screening adolescents for suicide risk, the regulation of e-cigarette advertising, and the adoption of educational programs that address the adverse effects of e-cigarettes. Moreover, this study’s results suggest that both current and previous use of e-cigarettes is associated with suicidal behaviors among adolescents; therefore, e-cigarette use should not be overlooked.

### Limitations

This study has limitations. We could not identify the direction of the associations or establish causality, although appropriate methods were used to mitigate these limitations. Furthermore, the KYRBWS data were collected anonymously online and self-reported, which may have produced nonrandom misclassification. We could not evaluate the concentration levels of nicotine or other e-liquid components or the dose-response relationship because the KYRBWS did not assess that information. The possibility of residual confounding also cannot be eliminated. In particular, although previous exposure to secondhand smoke may be associated with the initiation of smoking or suicidal behaviors, the KYRBWS did not include variables that could be used to fully assess the respondents’ history of exposure to secondhand smoke. The subgroup analyses showed significant associations even after adjusting for multiple comparisons through Bonferroni correction; however, some associations had wide 95% CIs owing to the small sample.

## Conclusions

In this cross-sectional study, adolescents who started smoking using e-cigarettes had a higher risk of suicidal behavior than did those who started smoking using conventional cigarettes. In addition, adolescents who initially used e-cigarettes and changed to conventional cigarettes were more likely to exhibit suicidal behaviors than were those who initially used conventional cigarettes and changed to e-cigarettes. These findings suggest that initial cigarette type is associated with suicidal behavior among adolescents and that consideration of initial cigarette type and subsequent change in cigarette type is warranted when conducting future research and formulating public policy.

## Notes

Supplement. **eTable 1.** Demographic Characteristics for Secondary Analyses of the Sample, 2015 to 2018 **eTable 2.** Association Between Initial Cigarette Type and Suicidal Behaviors Stratified by Current Smoking Status **eTable 3.** Sensitivity Analyses of the Association Between Cigarette Type Initially Smoked and Suicidal Behaviors **eTable 4.** Sensitivity Analyses of the Association Between Switching Cigarette Type and Suicidal Behaviors **eTable 5.** Sensitivity Analyses of the Association of the Timing of Switching Cigarette Type With Suicidal Behaviors

## References

[1] Cahn Z, Siegel M. Electronic cigarettes as a harm reduction strategy for tobacco control: a step forward or a repeat of past mistakes? J Public Health Policy. 2011;32(1):16-31. doi:10.1057/jphp.2010.41 21150942

[2] Vickerman KA, Carpenter KM, Altman T, Nash CM, Zbikowski SM. Use of electronic cigarettes among state tobacco cessation quitline callers. Nicotine Tob Res. 2013;15(10):1787-1791. doi:10.1093/ntr/ntt061 23658395

[3] Cullen KA, Ambrose BK, Gentzke AS, Apelberg BJ, Jamal A, King BA. Notes from the field: use of electronic cigarettes and any tobacco product among middle and high school students—United States, 2011-2018. MMWR Morb Mortal Wkly Rep. 2018;67(45):1276-1277. doi:10.15585/mmwr.mm6745a5 30439875 PMC6290807

[4] Korea Centers for Disease Control and Prevention. The statistics of 14th Korea Youth Risk Behavior Web-Based Survey. Korea Ministry of Education; 2018. Accessed October 19, 2020. http://www.kdca.go.kr

[5] Organization for Economic Cooperation and Development. Health at a glance 2019. OECD iLibrary; 2019. Accessed December 1, 2020. https://www.oecd-ilibrary.org/social-issues-migration-health/health-at-a-glance_19991312

[6] Korea National Statistical Office. Annual report on the causes of death statistics, 2017. Statistics Korea; 2017. Accessed December 1, 2020. https://kostat.go.kr/portal/eng/surveyOutline/5/1/index.static

[7] Korea National Statistical Office. Annual report on the causes of death statistics, 2018. Statistics Korea; 2018. Accessed December 1, 2020. https://kostat.go.kr/portal/eng/surveyOutline/5/1/index.static

[8] Nock MK, Borges G, Bromet EJ, Cha CB, Kessler RC, Lee S. Suicide and suicidal behavior. Epidemiol Rev. 2008;30(1):133-154. doi:10.1093/epirev/mxn002 18653727 PMC2576496

[9] Jacobs DG, Baldessarini RJ, Conwell Y, . Work Group on Suicidal Behaviors. Practice Guideline for the Assessment and Treatment of Patients With Suicidal Behaviors. American Psychiatric Association; 2010. Accessed December 13, 2020. https://psychiatryonline.org/pb/assets/raw/sitewide/practice_guidelines/guidelines/suicide.pdf

[10] Zhang L, Zhang D, Fang J, Wan Y, Tao F, Sun Y. Assessment of mental health of Chinese primary school students before and after school closing and opening during the COVID-19 pandemic. JAMA Netw Open. 2020;3(9):e2021482. doi:10.1001/jamanetworkopen.2020.21482 32915233 PMC7489803

[11] Brook JS, Schuster E, Zhang C. Cigarette smoking and depressive symptoms: a longitudinal study of adolescents and young adults. Psychol Rep. 2004;95(1):159-166. doi:10.2466/pr0.95.1.159-166 15460371

[12] Miller M, Hemenway D, Bell NS, Yore MM, Amoroso PJ. Cigarette smoking and suicide: a prospective study of 300,000 male active-duty army soldiers. Am J Epidemiol. 2000;151(11):1060-1063. doi:10.1093/oxfordjournals.aje.a010148 10873129

[13] Li D, Yang X, Ge Z, . Cigarette smoking and risk of completed suicide: a meta-analysis of prospective cohort studies. J Psychiatr Res. 2012;46(10):1257-1266. doi:10.1016/j.jpsychires.2012.03.013 22889465

[14] Poorolajal J, Darvishi N. Smoking and suicide: a meta-analysis. PLoS One. 2016;11(7):e0156348. doi:10.1371/journal.pone.0156348 27391330 PMC4938402

[15] Lange S, Koyanagi A, Rehm J, Roerecke M, Carvalho AF. Association of tobacco use and exposure to secondhand smoke with suicide attempts among adolescents: findings from 33 countries. Nicotine Tob Res. 2020;22(8):1322-1329. doi:10.1093/ntr/ntz172 31504808

[16] Leventhal AM, Strong DR, Sussman S, . Psychiatric comorbidity in adolescent electronic and conventional cigarette use. J Psychiatr Res. 2016;73:71-78. doi:10.1016/j.jpsychires.2015.11.008 26688438 PMC4738156

[17] Lee Y, Lee KS. Association of depression and suicidality with electronic and conventional cigarette use in South Korean adolescents. Subst Use Misuse. 2019;54(6):934-943. doi:10.1080/10826084.2018.1552301 30638103

[18] Kim JS, Kim K. Electronic cigarette use and suicidal behaviors among adolescents. J Public Health (Oxf). 2019;fdz086. doi:10.1093/pubmed/fdz086 31334765

[19] Chadi N, Li G, Cerda N, Weitzman ER. Depressive symptoms and suicidality in adolescents using e-cigarettes and marijuana: a secondary data analysis from the Youth Risk Behavior Survey. J Addict Med. 2019;13(5):362-365. doi:10.1097/ADM.0000000000000506 30688723

[20] Pham T, Williams JVA, Bhattarai A, Dores AK, Isherwood LJ, Patten SB. Electronic cigarette use and mental health: a Canadian population-based study. J Affect Disord. 2020;260:646-652. doi:10.1016/j.jad.2019.09.026 31542558

[21] Hua M, Talbot P. Potential health effects of electronic cigarettes: a systematic review of case reports. Prev Med Rep. 2016;4:169-178. doi:10.1016/j.pmedr.2016.06.002 27413679 PMC4929082

[22] Park EJ, Min YG. The emerging method of suicide by electronic cigarette liquid: a case report. J Korean Med Sci. 2018;33(11):e52. doi:10.3346/jkms.2018.33.e52 29495133 PMC5835582

[23] Kong G, Morean ME, Cavallo DA, Camenga DR, Krishnan-Sarin S. Reasons for electronic cigarette experimentation and discontinuation among adolescents and young adults. Nicotine Tob Res. 2015;17(7):847-854. doi:10.1093/ntr/ntu257 25481917 PMC4674436

[24] Best C, Haseen F, Currie D, . Relationship between trying an electronic cigarette and subsequent cigarette experimentation in Scottish adolescents: a cohort study. Tob Control. 2017;27(4):373-378. doi:10.1136/tobaccocontrol-2017-053691 28735273 PMC6047138

[25] Obisesan OH, Mirbolouk M, Osei AD, . Association between e-cigarette use and depression in the Behavioral Risk Factor Surveillance System, 2016-2017. JAMA Netw Open. 2019;2(12):e1916800. doi:10.1001/jamanetworkopen.2019.16800 31800073 PMC6902792

[26] Barrington-Trimis JL, Urman R, Berhane K, . E-cigarettes and future cigarette use. Pediatrics. 2016;138(1):e20160379. doi:10.1542/peds.2016-0379 27296866 PMC4925085

[27] Jeong W, Kim YK, Lee HJ, . Association of bedtime with both suicidal ideation and suicide planning among Korean adolescents. Int J Environ Res Public Health. 2019;16(20):3817. doi:10.3390/ijerph16203817 31658695 PMC6843598

[28] World Medical Association. World Medical Association Declaration of Helsinki: ethical principles for medical research involving human subjects. JAMA. 2013;310(20):2191-2194. doi:10.1001/jama.2013.281053 24141714

[29] von Elm E, Altman DG, Egger M, Pocock SJ, Gotzsche PC, Vandenbroucke JP; STROBE Initiative. The Strengthening the Reporting of Observational Studies in Epidemiology (STROBE) statement: guidelines for reporting observational studies. Epidemiology. 2007;18(6):800-804. doi:10.1097/EDE.0b013e3181577654 18049194

[30] Garcia de la Garza A, Blanco C, Olfson M, Wall MM. Identification of suicide attempt risk factors in a national US survey using machine learning. JAMA Psychiatry. Published online January 6, 2021. doi:10.1001/jamapsychiatry.2020.4165 33404590 PMC7788508

[31] Kwon JA, Lee M, Yoo KB, Park EC. Does the duration and time of sleep increase the risk of allergic rhinitis? results of the 6-year nationwide Korea Youth Risk Behavior Web-Based Survey. PLoS One. 2013;8(8):e72507. doi:10.1371/journal.pone.0072507 24015253 PMC3754987

[32] Hyeon JH, Shelley C, Lee CM. Prevalence and correlates of prior experimentation with e-cigarettes over conventional cigarettes among adolescents: findings from the 2015 Korea Youth Risk Behaviour Web-Based Survey. Tob Prev Cessat. 2019;5:33. doi:10.18332/tpc/112595 32411896 PMC7205116

[33] Goel R, Durand E, Trushin N, . Highly reactive free radicals in electronic cigarette aerosols. Chem Res Toxicol. 2015;28(9):1675-1677. doi:10.1021/acs.chemrestox.5b00220 26244921 PMC4961046

[34] Soneji S, Barrington-Trimis JL, Wills TA, . Association between initial use of e-cigarettes and subsequent cigarette smoking among adolescents and young adults: a systematic review and meta-analysis. JAMA Pediatr. 2017;171(8):788-797. doi:10.1001/jamapediatrics.2017.1488 28654986 PMC5656237

[35] Han MA, Kim KS, Ryu SY, Kang MG, Park J. Associations between smoking and alcohol drinking and suicidal behavior in Korean adolescents: Korea Youth Behavioral Risk Factor Surveillance, 2006. Prev Med. 2009;49(2-3):248-252. doi:10.1016/j.ypmed.2009.06.014 19573551

[36] Pagano T, Bida MR, Robinson RJ. Laboratory activity for the determination of nicotine in electronic cigarette liquids using gas chromatography–mass spectrometry. J Lab Chem Educ. 2015;3(3):37-43. 26478904 PMC4608496

[37] Miech R, Patrick ME, O’Malley PM, Johnston LD. What are kids vaping? results from a national survey of US adolescents. Tob Control. 2017;26(4):386-391. doi:10.1136/tobaccocontrol-2016-053014 27562412 PMC5326604

[38] Malone KM, Waternaux C, Haas GL, Cooper TB, Li S, Mann JJ. Cigarette smoking, suicidal behavior, and serotonin function in major psychiatric disorders. Am J Psychiatry. 2003;160(4):773-779. doi:10.1176/appi.ajp.160.4.773 12668368

[39] Jiang DG, Jin SL, Li GY, . Serotonin regulates brain-derived neurotrophic factor expression in select brain regions during acute psychological stress. Neural Regen Res. 2016;11(9):1471-1479. doi:10.4103/1673-5374.191222 27857753 PMC5090852

[40] Phillips B, Titz B, Kogel U, . Toxicity of the main electronic cigarette components, propylene glycol, glycerin, and nicotine, in Sprague-Dawley rats in a 90-day OECD inhalation study complemented by molecular endpoints. Food Chem Toxicol. 2017;109(Pt 1):315-332. doi:10.1016/j.fct.2017.09.001 28882640

[41] Lechner WV, Janssen T, Kahler CW, Audrain-McGovern J, Leventhal AM. Bi-directional associations of electronic and combustible cigarette use onset patterns with depressive symptoms in adolescents. Prev Med. 2017;96:73-78. doi:10.1016/j.ypmed.2016.12.034 28024859 PMC5510594

[42] Goniewicz ML, Kuma T, Gawron M, Knysak J, Kosmider L. Nicotine levels in electronic cigarettes. Nicotine Tob Res. 2013;15(1):158-166. doi:10.1093/ntr/nts103 22529223

[43] Kayir H, Semenova S, Markou A. Baseline impulsive choice predicts the effects of nicotine and nicotine withdrawal on impulsivity in rats. Prog Neuropsychopharmacol Biol Psychiatry. 2014;48:6-13. doi:10.1016/j.pnpbp.2013.09.007 24060391 PMC3858513

[44] Tam J, Warner KE. Students’ cigarette smoking and the perceived nicotine content of their e-cigarettes. Am J Prev Med. 2018;55(3):376-383. doi:10.1016/j.amepre.2018.04.034 30033026

[45] Leigh NJ, Lawton RI, Hershberger PA, Goniewicz ML. Flavourings significantly affect inhalation toxicity of aerosol generated from electronic nicotine delivery systems (ENDS). Tob Control. 2016;25(suppl 2):ii81-ii87. doi:10.1136/tobaccocontrol-2016-053205 27633767 PMC5784427

[46] Pandey GN, Rizavi HS, Ren X, . Proinflammatory cytokines in the prefrontal cortex of teenage suicide victims. J Psychiatr Res. 2012;46(1):57-63. doi:10.1016/j.jpsychires.2011.08.006 21906753 PMC3224201

[47] Zhao J, Nelson J, Dada O, Pyrgiotakis G, Kavouras IG, Demokritou P. Assessing electronic cigarette emissions: linking physico-chemical properties to product brand, e-liquid flavoring additives, operational voltage and user puffing patterns. Inhal Toxicol. 2018;30(2):78-88. doi:10.1080/08958378.2018.1450462 29564955 PMC6459014

[48] Gaur S, Agnihotri R. Health effects of trace metals in electronic cigarette aerosols—a systematic review. Biol Trace Elem Res. 2019;188(2):295-315. doi:10.1007/s12011-018-1423-x 29974385

[49] Badea M, Luzardo OP, Gonzalez-Antuna A, . Body burden of toxic metals and rare earth elements in non-smokers, cigarette smokers and electronic cigarette users. Environ Res. 2018;166:269-275. doi:10.1016/j.envres.2018.06.007 29908458

[50] Tobore TO. On the potential harmful effects of e-cigarettes (EC) on the developing brain: the relationship between vaping-induced oxidative stress and adolescent/young adults social maladjustment. J Adolesc. 2019;76:202-209. doi:10.1016/j.adolescence.2019.09.004 31574388

[51] Wang G, Liu W, Song W. Toxicity assessment of electronic cigarettes. Inhal Toxicol. 2019;31(7):259-273. doi:10.1080/08958378.2019.1671558 31556766

[52] Wills TA, Sargent JD, Gibbons FX, Pagano I, Schweitzer R. E-cigarette use is differentially related to smoking onset among lower risk adolescents. Tob Control. 2016;26(5):534-539. doi:10.1136/tobaccocontrol-2016-053116 27543564 PMC5537057

[53] Berry KM, Fetterman JL, Benjamin EJ, . Association of electronic cigarette use with subsequent initiation of tobacco cigarettes in US youths. JAMA Netw Open. 2019;2(2):e187794. doi:10.1001/jamanetworkopen.2018.7794 30707232 PMC6484602

[54] Grant JE, Lust K, Fridberg DJ, King AC, Chamberlain SR. E-cigarette use (vaping) is associated with illicit drug use, mental health problems, and impulsivity in university students. Ann Clin Psychiatry. 2019;31(1):27-35. doi:10.1080/10401230490281366 30699215 PMC6420081

[55] Al Rifai M, Mirbolouk M, Obisesan OH, . The association of electronic cigarette use and the subjective domains of physical and mental health: the Behavioral Risk Factor Surveillance System Survey. Cureus. 2020;12(2):e7088. doi:10.7759/cureus.7088 32226689 PMC7096000

