# Supplementary material for: Association of Cigarette Type Initially Smoked With Suicidal Behaviors Among Adolescents in Korea From 2015 to 2018
Source: JAMA Netw Open. 2021 Apr 30;4(4):e218803. doi: 10.1001/jamanetworkopen.2021.8803 (PMC8087956; doi:10.1001/jamanetworkopen.2021.8803)
Supplement: Supplement. — eTable 1. Demographic Characteristics for Secondary Analyses of the Sample, 2015 to 2018 eTable 2. Association Between Initial Cigarette Type and Suicidal Behaviors Stratified by Current Smoking Status eTable 3. Sensitivity Analyses of the Association Between Cigarette Type Initially Smoked and Suicidal Behaviors eTable 4. Sensitivity Analyses of the Association Between Switching Cigarette Type and Suicidal Behaviors eTable 5. Sensitivity Analyses of the Association of the Timing of Switching Cigarette Type With Suicidal Behaviors [file jamanetwopen-e218803-s001.pdf]

Supplementary Online Content

Kim SH, Jeong SH, Park EC, Jang SI. Association of cigarette type initially smoked with suicidal behaviors among adolescents in Korea from 2015 to 2018. *JAMA Netw Open*. 2021;4(4):e218803. doi:10.1001/jamanetworkopen.2021.8803

eTable 1. Demographic Characteristics for Secondary Analyses of the Sample, 2015 to 2018

eTable 2. Association Between Initial Cigarette Type and Suicidal Behaviors Stratified by Current Smoking Status

eTable 3. Sensitivity Analyses of the Association Between Cigarette Type Initially Smoked and Suicidal Behaviors

eTable 4. Sensitivity Analyses of the Association Between Switching Cigarette Type and Suicidal Behaviors

eTable 5. Sensitivity Analyses of the Association of the Timing of Switching Cigarette Type With Suicidal Behaviors

This supplementary material has been provided by the authors to give readers additional information about their work.

© 2021 Kim SH et al. *JAMA Network Open*.

eTable 1. Demographic Characteristics for Secondary Analyses of the Sample, 2015 to 2018

	Participants													
	Total	Never smoked		Used Only c-cig.		Used Only e-cig.		Switched from c-cig. to e-cig. (c-cig.→e-cig.)[a]		Initiated smoking using both e-cig. & c-cig. (in same year)		Switched from e-cig. to c-cig. (e-cig.→c-cig.)[a]		
**Characteristics**	**N**	**N**	**%**	**N**	**%**	**N**	**%**	**N**	**%**	**N**	**%**	**N**	**%**	**P-value**
**Male (n=131,094)**	**131,094**	**100,416**	**76.6**	**13,886**	**10.59**	**2,412**	**1.8**	**9,464**	**7.2**	**4,018**	**3.1**	**898**	**0.7**	
**School grade**														
7	21,230	19,931	93.9	859	4.0	183	0.9	86	0.4	147	0.7	24	0.1	<.001
8	21,913	18,971	86.6	1,576	7.2	282	1.3	440	2.0	538	2.5	106	0.5	
9	22,437	17,808	79.4	2,224	9.9	443	2.0	998	4.4	792	3.5	172	0.8	
10	21,520	15,387	71.5	2,629	12.2	497	2.3	1,901	8.8	906	4.2	200	0.9	
11	22,241	14,758	66.4	3,139	14.1	530	2.4	2,721	12.2	901	4.1	192	0.9	
12	21,753	13,561	62.3	3,459	15.9	477	2.2	3,318	15.3	734	3.4	204	0.9	
**Economic status**														
Low	3,928	2,327	59.2	618	15.7	115	2.9	581	14.8	226	5.8	61	1.6	<.001
Medium-low	15,491	10,926	70.5	2,098	13.5	298	1.9	1,540	9.9	515	3.3	114	0.7	
Medium	57,982	44,580	76.9	6,339	10.9	1,012	1.7	4,053	7.0	1,678	2.9	320	0.6	
Medium-high	37,800	30,103	79.6	3,437	9.1	658	1.7	2,312	6.1	1,052	2.8	238	0.6	
High	15,893	12,480	78.5	1,394	8.8	329	2.1	978	6.2	547	3.4	165	1.0	
**Living situation**														
Without extended or immediate family	5,962	4,050	67.9	827	13.9	181	3.0	518	8.7	288	4.8	98	1.6	<.001
With extended family	1,131	674	59.6	168	14.9	54	4.8	132	11.7	56	5.0	47	4.2	
With immediate family	124,001	95,692	77.2	12,891	10.4	2,177	1.8	8,814	7.1	3,674	3.0	753	0.6	
**Academic achievement**														
Low	14,292	8,688	60.8	2,112	14.8	350	2.4	2,173	15.2	798	5.6	171	1.2	<.001

© 2021 Kim SH et al. *JAMA Network Open*.

Medium-low	28,883	20,670	71.6	3,697	12.8	588	2.0	2,698	9.3	1,001	3.5	229	0.8	
Medium	36,011	28,152	78.2	3,703	10.3	642	1.8	2,310	6.4	1,003	2.8	201	0.6	
Medium-high	32,233	26,507	82.2	2,850	8.8	491	1.5	1,479	4.6	754	2.3	152	0.5	
High	19,675	16,399	83.3	1,524	7.7	341	1.7	804	4.1	462	2.3	145	0.7	
**Alcohol use**														
Ever	57,494	32,742	56.9	10,213	17.8	1,624	2.8	8,794	15.3	3,361	5.8	760	1.3	<.001
Never	73,600	67,674	91.9	3,673	5.0	788	1.1	670	0.9	657	0.9	138	0.2	
**Physical activity level**														
Low	53,224	41,299	77.6	5,583	10.5	820	1.5	3,828	7.2	1,403	2.6	291	0.5	<.001
High	77,870	59,117	75.9	8,303	10.7	1,592	2.0	5,636	7.2	2,615	3.4	607	0.8	
**Self-reported health status**														
Low	6,515	4,744	72.8	773	11.9	105	1.6	630	9.7	211	3.2	52	0.8	<.001
Medium	23,570	17,770	75.4	2,667	11.3	423	1.8	1,836	7.8	712	3.0	162	0.7	
High	101,009	77,902	77.1	10,446	10.3	1,884	1.9	6,998	6.9	3,095	3.1	684	0.7	
**Perceived stress level**														
Low	33,654	27,154	80.7	3,051	9.1	542	1.6	1,836	5.5	862	2.6	209	0.6	<.001
Medium	57,708	44,586	77.3	6,081	10.5	1,047	1.8	3,945	6.8	1,689	2.9	360	0.6	
High	39,732	28,676	72.2	4,754	12.0	823	2.1	3,683	9.3	1,467	3.7	329	0.8	
**Suicidal ideation**														
Yes	12,271	8,062	65.7	1,735	14.1	308	2.5	1,339	10.9	635	5.2	192	1.6	<.001
No	118,823	92,354	77.7	12,151	10.2	2,104	1.8	8,125	6.8	3,383	2.8	706	0.6	
**Suicide planning**														
Yes	4,615	2,867	62.1	664	14.4	163	3.5	527	11.4	286	6.2	108	2.3	<.001
No	126,479	97,549	77.1	13,222	10.5	2,249	1.8	8,937	7.1	3,732	3.0	790	0.6	
**Suicide attempt**														
Yes	2,613	1,489	57.0	375	14.4	103	3.9	383	14.7	188	7.2	75	2.9	<.001

© 2021 Kim SH et al. *JAMA Network Open*.

No	128,481	98,927	77.0	13,511	10.5	2,309	1.8	9,081	7.1	3,830	3.0	823	0.6	
**Female (n=124,793)**	**124,793**	**114,545**	**91.8**	**6,313**	**5.06**	**685**	**0.5**	**2,058**	**1.6**	**925**	**0.7**	**267**	**0.2**	
**School grade**														
7	20,075	19,620	97.7	295	1.5	67	0.3	23	0.1	54	0.3	16	0.1	<.001
8	20,515	19,483	95.0	651	3.2	89	0.4	117	0.6	141	0.7	34	0.2	
9	21,462	19,850	92.5	1,018	4.7	124	0.6	253	1.2	179	0.8	38	0.2	
10	20,382	18,428	90.4	1,187	5.8	129	0.6	391	1.9	197	1.0	50	0.2	
11	20,781	18,319	88.2	1,485	7.1	141	0.7	585	2.8	186	0.9	65	0.3	
12	21,578	18,845	87.3	1,677	7.8	135	0.6	689	3.2	168	0.8	64	0.3	
**Economic status**														
Low	3,233	2,511	77.7	371	11.5	37	1.1	186	5.8	75	2.3	53	1.6	<.001
Medium-low	16,044	14,003	87.3	1,265	7.9	109	0.7	448	2.8	164	1.0	55	0.3	
Medium	61,426	56,879	92.6	2,937	4.8	288	0.5	883	1.4	362	0.6	77	0.1	
Medium-high	34,283	32,120	93.7	1,351	3.9	161	0.5	390	1.1	215	0.6	46	0.1	
High	9,807	9,032	92.1	389	4.0	90	0.9	151	1.5	109	1.1	36	0.4	
**Living situation**														
Without extended or immediate family	5,074	4,419	87.1	331	6.5	45	0.9	133	2.6	84	1.7	62	1.2	<.001
With extended family	848	622	73.3	83	9.8	34	4.0	64	7.5	19	2.2	26	3.1	
With immediate family	118,871	109,504	92.1	5,899	5.0	606	0.5	1,861	1.6	822	0.7	179	0.2	
**Academic achievement**														
Low	11,671	9,374	80.3	1,248	10.7	117	1.0	627	5.4	233	2.0	72	0.6	<.001
Medium-low	29,254	26,278	89.8	1,873	6.4	186	0.6	603	2.1	254	0.9	60	0.2	
Medium	36,903	34,524	93.6	1,580	4.3	147	0.4	391	1.1	200	0.5	61	0.2	
Medium-high	32,739	30,981	94.6	1,173	3.6	122	0.4	293	0.9	136	0.4	34	0.1	
High	14,226	13,388	94.1	439	3.1	113	0.8	144	1.0	102	0.7	40	0.3	
**Alcohol use**														

© 2021 Kim SH et al. *JAMA Network Open*.

Ever	43,625	34,778	79.7	5,281	12.1	482	1.1	1,992	4.6	851	2.0	241	0.6	<.001
Never	81,168	79,767	98.3	1,032	1.3	203	0.3	66	0.1	74	0.1	26	0.0	
**Physical activity level**														
Low	84,362	77,567	91.9	4,335	5.1	405	0.5	1,368	1.6	562	0.7	125	0.1	<.001
High	40,431	36,978	91.5	1,978	4.9	280	0.7	690	1.7	363	0.9	142	0.4	
**Self-reported health status**														
Low	9,306	8,005	86.0	768	8.3	66	0.7	322	3.5	113	1.2	32	0.3	<.001
Medium	31,493	28,414	90.2	1,910	6.1	182	0.6	661	2.1	256	0.8	70	0.2	
High	83,994	78,126	93.0	3,635	4.3	437	0.5	1,075	1.3	556	0.7	165	0.2	
**Perceived stress level**														
Low	17,579	16,563	94.2	584	3.3	86	0.5	181	1.0	110	0.6	55	0.3	<.001
Medium	51,071	47,878	93.7	2,061	4.0	221	0.4	540	1.1	286	0.6	85	0.2	
High	56,143	50,104	89.2	3,668	6.5	378	0.7	1,337	2.4	529	0.9	127	0.2	
**Suicidal ideation**														
Yes	18,996	16,082	84.7	1,702	9.0	178	0.9	634	3.3	299	1.6	101	0.5	<.001
No	105,797	98,463	93.1	4,611	4.4	507	0.5	1,424	1.3	626	0.6	166	0.2	
**Suicide planning**														
Yes	5,720	4,534	79.3	624	10.9	83	1.5	274	4.8	142	2.5	63	1.1	<.001
No	119,073	110,011	92.4	5,689	4.8	602	0.5	1,784	1.5	783	0.7	204	0.2	
**Suicide attempt**														
Yes	4,086	3,041	74.4	531	13.0	76	1.9	248	6.1	132	3.2	58	1.4	<.001
No	120,707	111,504	92.4	5,782	4.8	609	0.5	1,810	1.5	793	0.7	209	0.2	

[a] Including all participants who smoked the other cigarette type after the first smoking.
Abbreviations: e-cigarettes, electronic cigarettes; c-cigarettes, conventional cigarettes.

© 2021 Kim SH et al. *JAMA Network Open*.

eTable 2. Association Between Initial Cigarette Type and Suicidal Behaviors Stratified by Current Smoking Status

Variables		Suicidal ideation			Suicide planning			Suicide attempt		
**Current smoking status**	**Initial cigarette type**	**AOR**	**95% CI**	***P*-value**	**AOR**	**95% CI**	***P*-value**	**AOR**	**95% CI**	***P*-value**
**Male**										
Dual user	Started with e-cig.	3.87	(2.75 – 5.43)	<.001[a]	6.23	(4.35 – 8.91)	<.001[a]	7.08	(4.83 – 10.36)	<.001[a]
	Started with c-cig.	1.91	(1.71 – 2.13)	<.001[a]	2.67	(2.31 – 3.07)	<.001[a]	3.56	(3.03 – 4.19)	<.001[a]
E-cig. user	Started with e-cig.	2.68	(2.08 – 3.44)	<.001[a]	4.18	(3.21 – 5.44)	<.001[a]	5.56	(4.07 – 7.61)	<.001[a]
	Started with c-cig.	1.28	(0.94 – 1.74)	.11	1.67	(1.09 – 2.55)	.02	2.15	(1.27 – 3.64)	.004
C-cig. user	Started with e-cig.	2.30	(1.56 – 3.38)	<.001[a]	2.63	(1.52 – 4.55)	.001[a]	2.87	(1.35 – 6.09)	.01
	Started with c-cig.	1.41	(1.30 – 1.54)	<.001[a]	1.74	(1.53 – 1.98)	<.001[a]	2.14	(1.83 – 2.51)	<.001[a]
Ex-smoker	Started with e-cig.	1.23	(1.05 – 1.44)	.01	1.57	(1.25 – 1.98)	.001[a]	1.52	(1.12 – 2.07)	.01
	Started with c-cig.	1.24	(1.16 – 1.32)	<.001[a]	1.19	(1.07 – 1.33)	<.001[a]	1.29	(1.11 – 1.49)	<.001[a]
Never smoked		**1.00**			**1.00**			**1.00**		
**Female**										
Dual user	Started with e-cig.	4.70	(2.78 – 7.96)	<.001[a]	6.85	(4.03 – 11.62)	<.001[a]	11.04	(6.21 – 19.63)	<.001[a]
	Started with c-cig.	2.34	(1.97 – 2.78)	<.001[a]	3.67	(2.99 – 4.51)	<.001[a]	5.15	(4.15 – 6.38)	<.001[a]
E-cig. user	Started with e-cig.	2.46	(1.61 – 3.76)	<.001[a]	5.16	(3.24 – 8.21)	<.001[a]	5.41	(3.21 – 9.13)	<.001[a]
	Started with c-cig.	1.99	(1.14 – 3.47)	.02	4.55	(2.22 – 9.34)	<.001[a]	3.79	(1.97 – 7.28)	<.001[a]
C-cig. user	Started with e-cig.	1.45	(0.75 – 2.81)	.27	2.58	(1.19 – 5.62)	.02	3.99	(1.92 – 8.32)	.001[a]
	Started with c-cig.	1.65	(1.48 – 1.83)	<.001[a]	2.17	(1.88 – 2.50)	<.001[a]	2.85	(2.47 – 3.29)	<.001[a]
Ex-smoker	Started with e-cig.	1.36	(1.07 – 1.74)	.01	1.68	(1.20 – 2.37)	.003	2.54	(1.82 – 3.55)	<.001[a]
	Started with c-cig.	1.36	(1.25 – 1.48)	<.001[a]	1.44	(1.27 – 1.62)	<.001[a]	1.81	(1.59 – 2.06)	<.001[a]
Never smoked		**1.00**			**1.00**			**1.00**		

© 2021 Kim SH et al. *JAMA Network Open*.

Adjusted for school grade, economic status, living situation, academic achievement, alcohol use, physical activity level, self-reported health status, perceived stress level.
[a] Statistically significant after applying Bonferroni correction.
Abbreviations: AOR, adjusted odds ratio; e-cig., electronic cigarettes; c-cig., conventional cigarettes.

© 2021 Kim SH et al. *JAMA Network Open*.

eTable 3. Sensitivity Analyses of the Association Between Cigarette Type Initially Smoked and Suicidal Behaviors

Variables	Suicidal ideation						Suicide planning						Suicide attempt					
	Male			Female			Male			Female			Male			Female		
	AOR	95% CI	*P*-value	AOR	95% CI	*P*-value	AOR	95% CI	*P*-value	AOR	95% CI	*P*-value	AOR	95% CI	*P*-value	AOR	95% CI	*P*-value
**Sensitivity analysis (1) - excluding those who started smoking or changed the cigarette type within 12 months from the time of survey [a]**																		
**Never smoking as reference**																		
Started with e-cigarette	1.71	(1.49–1.96)	<.001[c]	1.89	(1.54–2.32)	<.001[c]	2.68	(2.26–3.18)	<.001[c]	3.16	(2.48–4.02)	<.001[c]	3.07	(2.49–3.77)	<.001[c]	4.66	(3.63–5.99)	<.001[c]
Started with c-cigarette	1.35	(1.27–1.43)	<.001[c]	1.48	(1.38–1.59)	<.001[c]	1.51	(1.38–1.65)	<.001[c]	1.89	(1.71–2.03)	<.001[c]	1.85	(1.65–2.06)	<.001[c]	2.43	(2.19–2.70)	<.001[c]
Never smoked	**1.00**			**1.00**			**1.00**			**1.00**			**1.00**			**1.00**		
**Started with c-cigarette as reference**																		
Started with e-cigarette	1.27	(1.11–1.46)	<.001[c]	1.28	(1.03–1.58)	.03	1.78	(1.49–2.12)	<.001[c]	1.67	(1.30–2.15)	<.001[c]	1.66	(1.35–2.05)	<.001[c]	1.92	(1.48–2.50)	<.001[c]
Never smoked	0.74	(0.70–0.79)	<.001[c]	0.68	(0.63–0.72)	<.001[c]	0.66	(0.61–0.72)	<.001[c]	0.53	(0.48–0.58)	<.001[c]	0.54	(0.48–0.61)	<.001[c]	0.41	(0.37–0.45)	<.001[c]
Started with c-cigarette	**1.00**			**1.00**			**1.00**			**1.00**			**1.00**			**1.00**		
**Sensitivity analysis (2) - age as a covariate [b]**																		
**Never smoking as reference**																		
Started with e-cigarette	1.69	(1.49–1.92)	<.001[c]	1.66	(1.36–2.02)	<.001[c]	2.39	(2.04–2.80)	<.001[c]	2.37	(1.83–3.06)	<.001[c]	2.69	(2.20–3.30)	<.001[c]	3.60	(2.79–4.65)	<.001[c]
Started with c-cigarette	1.35	(1.27–1.42)	<.001[c]	1.51	(1.41–1.61)	<.001[c]	1.47	(1.35–1.61)	<.001[c]	1.79	(1.63–1.97)	<.001[c]	1.76	(1.58–1.97)	<.001[c]	2.30	(2.08–2.54)	<.001[c]
Never smoked	**1.00**			**1.00**			**1.00**			**1.00**			**1.00**			**1.00**		
**Started with c-cigarette as reference**																		
Started with e-cigarette	1.26	(1.11–1.43)	<.001[c]	1.10	(0.90–1.35)	.36	1.63	(1.38–1.91)	<.001[c]	1.32	(1.02–1.72)	.03	1.53	(1.25–1.87)	<.001[c]	1.57	(1.20–2.05)	<.001[c]
Never smoked	0.74	(0.70–0.79)	<.001[c]	0.66	(0.62–0.71)	<.001[c]	0.68	(0.62–0.74)	<.001[c]	0.56	(0.51–0.61)	<.001[c]	0.57	(0.51–0.64)	<.001[c]	0.44	(0.39–0.48)	<.001[c]
Started with c-cigarette	**1.00**			**1.00**			**1.00**			**1.00**			**1.00**			**1.00**		
[a] Included sample: total, 249,395; male, 126,412; female, 122,983.																		

© 2021 Kim SH et al. *JAMA Network Open*.

[b] Included sample: total, 254,478; male, 130,184; female, 124,294.<br>[c] Statistically significant after applying Bonferroni correction.
Adjusted for school grade[a] or age[b], economic status, living situation, academic achievement, alcohol use, physical activity level, self-reported health status, perceived stress level.
Abbreviations: AOR, adjusted odds ratio; e-cigarette, electronic cigarettes; c-cigarette, conventional cigarettes.

© 2021 Kim SH et al. *JAMA Network Open*.

eTable 4. Sensitivity Analyses of the Association Between Switching Cigarette Type and Suicidal Behaviors

Variables	Suicidal ideation						Suicide planning						Suicide attempt					
	Male			Female			Male			Female			Male			Female		
	AOR	95% CI	*P*-value	AOR	95% CI	*P*-value	AOR	95% CI	*P*-value	AOR	95% CI	*P*-value	AOR	95% CI	*P*-value	AOR	95% CI	*P*-value
**Sensitivity analysis (1) - excluding those who started smoking or changed the cigarette type within 12 months from the time of survey [a]**																		
**Never smoking as reference**																		
Switched from e-cig. to c-cig. **(e-cig. → c-cig. or both)**	2.49	(1.94–3.19)	<.001[c]	2.96	(1.90–4.61)	<.001[c]	3.94	(2.94–5.29)	<.001[c]	4.80	(3.10–7.44)	<.001[c]	4.39	(3.16–6.10)	<.001[c]	7.92	(5.00–12.54)	<.001[c]
Initiated smoking using both e-cig. & c-cig. **(in same year)**	1.59	(1.42–1.78)	<.001[c]	1.90	(1.57–2.29)	<.001[c]	1.96	(1.68–2.29)	<.001[c]	2.86	(2.26–3.63)	<.001[c]	2.48	(2.06–2.98)	<.001[c]	3.86	(3.00–4.97)	<.001[c]
Switched from c-cig. to e-cig. **(c-cig. → e-cig. or both)**	1.34	(1.22–1.47)	<.001[c]	1.45	(1.26–1.67)	<.001[c]	1.49	(1.31–1.71)	<.001[c]	2.11	(1.76–2.53)	<.001[c]	2.11	(1.80–2.48)	<.001[c]	2.95	(2.44–3.57)	<.001[c]
Used only e-cig.	1.47	(1.26–1.72)	<.001[c]	1.59	(1.24–2.04)	<.001[c]	2.24	(1.83–2.75)	<.001[c]	2.62	(1.96–3.50)	<.001[c]	2.61	(2.02–3.36)	<.001[c]	3.66	(2.69–4.98)	<.001[c]
Used only c-cig.	1.29	(1.20–1.38)	<.001[c]	1.44	(1.32–1.56)	<.001[c]	1.40	(1.25–1.57)	<.001[c]	1.69	(1.53–1.91)	<.001[c]	1.52	(1.31–1.76)	<.001[c]	2.08	(1.83–2.37)	<.001[c]
Never smoked	**1.00**			**1.00**			**1.00**			**1.00**			**1.00**			**1.00**		
**Switching from c-cig. to e-cig. as reference**																		
Switched from e-cig. to c-cig. **(e-cig. → c-cig. or both)**	1.86	(1.44–2.41)	<.001[c]	2.04	(1.30–3.23)	.002[c]	2.64	(1.95–3.57)	<.001[c]	2.27	(1.43–3.62)	<.001[c]	2.08	(1.47–2.94)	<.001[c]	2.68	(1.64–4.40)	<.001[c]
Initiated smoking using both e-cig. & c-cig. **(in same year)**	1.19	(1.04–1.36)	.01	1.31	(1.03–1.66)	.03	1.31	(1.09–1.57)	<.001	1.36	(1.01–1.82)	.04	1.17	(0.95–1.45)	.14	1.31	(0.96–1.77)	.09
Switched from c-cig. to e-cig. **(c-cig. → e-cig. or both)**	**1.00**			**1.00**			**1.00**			**1.00**			**1.00**			**1.00**		
**Sensitivity analysis (2) - age as a covariate [b]**																		
**Never smoking as reference**																		

© 2021 Kim SH et al. *JAMA Network Open*.

Switched from e-cig. to c-cig. **(e-cig. → c-cig. or both)**	2.55	(2.05–3.18)	<.001[c]	2.52	(1.64–3.86)	<.001[c]	3.39	(2.60–4.43)	<.001[c]	3.60	(2.25–5.75)	<.001[c]	3.94	(2.89–5.37)	<.001[c]	6.75	(4.25–10.73)	<.001[c]
Initiated smoking using both e-cig. & c-cig. **(in same year)**	1.53	(1.37–1.70)	<.001[c]	1.82	(1.53–2.16)	<.001[c]	1.71	(1.47–2.00)	<.001[c]	2.47	(1.96–3.12)	<.001[c]	2.09	(1.72–2.53)	<.001[c]	3.07	(2.40–3.93)	<.001[c]
Switched from c-cig. to e-cig. **(c-cig. → e-cig. or both)**	1.33	(1.21–1.45)	<.001[c]	1.44	(1.27–1.63)	<.001[c]	1.82	(1.30–1.68)	<.001[c]	1.85	(1.56–2.19)	<.001[c]	2.02	(1.73–2.37)	<.001[c]	2.61	(2.18–3.11)	<.001[c]
Used only e-cig.	1.40	(1.21–1.63)	<.001[c]	1.44	(1.15–1.81)	.002[c]	2.01	(1.65–2.44)	<.001[c]	1.98	(1.45–2.69)	<.001[c]	2.22	(1.72–2.87)	<.001[c]	2.69	(1.94–3.73)	<.001[c]
Used only c-cig.	1.31	(1.23–1.41)	<.001[c]	1.49	(1.38–1.61)	<.001[c]	1.40	(1.26–1.57)	<.001[c]	1.68	(1.50–1.87)	<.001[c]	1.52	(1.32–1.76)	<.001[c]	2.09	(1.85–2.35)	<.001[c]
Never smoked	**1.00**			**1.00**			**1.00**			**1.00**			**1.00**			**1.00**		
**Switching from c-cig. to e-cig. as reference**																		
Switched from e-cig. to c-cig. **(e-cig. → c-cig. or both)**	1.92	(1.53–2.42)	<.001[c]	1.75	(1.13–2.71)	.008	2.29	(1.74–3.02)	<.001[c]	1.94	(1.19–3.19)	.003	1.95	(1.41–2.70)	<.001[c]	2.59	(1.59–4.24)	<.001[c]
Initiated smoking using both e-cig. & c-cig. **(in same year)**	1.15	(1.02–1.31)	.03	1.26	(1.02–1.56)	.03	1.16	(0.97–1.37)	.09	1.34	(1.01–1.77)	.04	1.03	(0.83–1.29)	.71	1.18	(0.88–1.57)	.24
Switched from c-cig. to e-cig. **(c-cig. → e-cig. or both)**	**1.00**			**1.00**			**1.00**			**1.00**			**1.00**			**1.00**		

[a] Included sample: total, 249,395; male, 126,412; female, 122,983.

[b] Included sample: total, 254,478; male, 130,184; female, 124,294.

[c] Statistically significant after applying Bonferroni correction.

Adjusted for school grade[a] or age[b], economic status, living situation, academic achievement, alcohol use, physical activity level, self-reported health status, perceived stress level.

Abbreviations: AOR, adjusted odds ratio; e-cig., electronic cigarettes; c-cig., conventional cigarettes.

© 2021 Kim SH et al. *JAMA Network Open*.

eTable 5. Sensitivity Analyses of the Association of the Timing of Switching Cigarette Type With Suicidal Behaviors

Variables	Suicidal ideation						Suicide planning					
	Male participants			Female participants			Male participants			Female participants		
	AOR	95% CI	*P*-value	AOR	95% CI	*P*-value	AOR	95% CI	*P*-value	AOR	95% CI	*P*-value
**Sensitivity analysis (1) - excluding those who started smoking or changed the cigarette type within 12 months from the time of survey** [a]												
**Timing of the switching cigarette type**												
Started with e-cig. & switched after 2 years	3.93	(2.57 – 5.99)	<.001[c]	4.08	(1.99 – 8.39)	<.001[c]	5.53	(3.48 – 8.76)	<.001[c]	8.55	(4.48 – 16.32)	<.001[c]
Started with e-cig. & switched in 2 years	2.00	(1.48 – 2.70)	<.001[c]	2.43	(1.42 – 4.18)	.002[c]	3.29	(2.27 – 4.75)	<.001[c]	3.09	(1.67 – 5.70)	<.001[c]
Started with c-cig. & switched after 2 years	1.30	(1.14 – 1.49)	<.001[c]	1.40	(1.10 – 1.77)	.005	1.52	(1.25 – 1.84)	<.001[c]	1.98	(1.46 – 2.68)	<.001[c]
Started with c-cig. & switched in 2 years	1.36	(1.22 – 1.52)	<.001[c]	1.48	(1.24 – 1.76)	<.001[c]	1.49	(1.26 – 1.75)	<.001[c]	2.20	(1.76 – 2.74)	<.001[c]
Started with both e-cig. & c-cig. in same time (e-cig. = c-cig.)	1.59	(1.42 – 1.78)	<.001[c]	1.90	(1.57 – 2.29)	<.001[c]	1.96	(1.68 – 2.29)	<.001[c]	2.87	(2.26 – 3.64)	<.001[c]
Used only one cigarette type	1.31	(1.23 – 1.40)	<.001[c]	1.45	(1.34 – 1.57)	<.001[c]	1.52	(1.37 – 1.68)	<.001[c]	1.77	(1.58 – 1.98)	<.001[c]
Never smoked	**1.00**			**1.00**			**1.00**			**1.00**		
**Sensitivity analysis (2) - age as a covariate** [b]												
**Timing of the switching cigarette type**												
Started with e-cig. & switched after 2 years	4.32	(2.86 – 6.51)	<.001[c]	3.03	(1.36 – 6.77)	.006	5.98	(3.87 – 9.25)	<.001[c]	6.09	(2.85 – 13.01)	<.001[c]
Started with e-cig. & switched in 2 years	2.07	(1.60 – 2.68)	<.001[c]	2.32	(1.43 – 3.76)	.001[c]	2.56	(1.83 – 3.58)	<.001[c]	2.70	(1.46 – 4.98)	.002[c]
Started with c-cig. & switched after 2 years	1.29	(1.13 – 1.46)	.002[c]	1.33	(1.08 – 1.63)	.02	1.51	(1.27 – 1.80)	<.001[c]	1.75	(1.34 – 2.28)	<.001[c]
Started with c-cig. & switched in 2 years	1.35	(1.23 – 1.49)	<.001[c]	1.52	(1.31 – 1.76)	<.001[c]	1.47	(1.26 – 1.71)	<.001[c]	1.92	(1.56 – 2.37)	<.001[c]
Started with both e-cig. & c-cig. in same time (e-cig. = c-cig.)	1.53	(1.37 – 1.70)	<.001[c]	1.82	(1.53 – 2.16)	<.001[c]	1.71	(1.47 – 2.00)	<.001[c]	2.47	(1.96 – 3.12)	<.001[c]
Used only one cigarette type	1.33	(1.24 – 1.41)	<.001[c]	1.49	(1.38 – 1.60)	<.001[c]	1.49	(1.35 – 1.65)	<.001[c]	1.70	(1.53 – 1.89)	<.001[c]

© 2021 Kim SH et al. *JAMA Network Open*.

Never smoked	**1.00**						**1.00**						**1.00**						**1.00**					

[a] Included sample: total, 249,395; male, 126,412; female, 122,983.

[b] Included sample: total, 254,478; male, 130,184; female, 124,294.

[c] Statistically significant after applying Bonferroni correction.

Adjusted for school grade[a] or age[b], economic status, living situation, academic achievement, alcohol use, physical activity level, self-reported health status, perceived stress level.

Abbreviations: AOR, adjusted odds ratio; e-cig., electronic cigarettes; c-cig., conventional cigarettes.

© 2021 Kim SH et al. *JAMA Network Open*.
